# Ending the Social Normalization of Violence against Children in Canada: A Framework, Rationale, and Appeal to Canadian Faith Leaders

**DOI:** 10.3390/ijerph192417016

**Published:** 2022-12-18

**Authors:** Valerie Michaelson, Ron Ensom

**Affiliations:** 1 Department of Health Sciences, Faculty of Applied Health Sciences, Brock University, St. Catharines, ON L2S 3A1, Canada; 2 Children’s Hospital of Eastern Ontario, Ottawa, ON K1H 8L1, Canada

**Keywords:** parenting, prevention strategies, child maltreatment, child protection, social norms, religion, church

## Abstract

Corporal punishment remains the most common form of violence against children in Canada. Along with being legally permitted, it is made socially acceptable through cultural and social norms often disguised as discipline. Paraphrases of Judeo-Christian sacred texts such as “spare the rod; spoil the child” have been widely used to justify corporal punishment, and to create norms around the idea that it is a god-given responsibility of parents to inflict corporal punishment on their children “for their own good.” Corporal punishment is then not only an acceptable part of raising children but can be a godly duty. Though Canada is a secular country, this social norm provides a moral hegemony under which violence becomes the status quo and has proven very difficult to change. In this commentary, we outline the ways that Christian religion has contributed to social norms around corporal punishment. We then argue that religious leaders have an opportunity—and moral responsibility—to contribute to change. We conclude with insights from social norms theory and offer evidence-based recommendations for ways forward that could help shift attitudes around corporal punishment in order to decrease its prevalence and harm. While each of these issues has been written about extensively in isolation, this commentary offers an analysis of these matters together under one umbrella. By making this relationship between Christianity and the social norms that propagate corporal punishment more visible, we draw attention to the role leaders in Christian churches could play in disrupting the social acceptance of violence against our youngest Canadian citizens. We provide a practical and evidence-based framework, rationale, and appeal to Canadian faith leaders to use their influence to add momentum to a critical health, rights—and religion—issue in Canada.

## 1. Introduction

Corporal punishment (also known as physical punishment) involves an action intended to cause physical discomfort or pain to correct a child’s behaviour, to “teach a lesson”, or to deter the child from repeating a behaviour [1]. A compelling body of research evidence demonstrates the serious health and developmental threat that it poses to children [2,3,4]. For example, corporal punishment predicts increased child aggression, mental health problems, substance use, depression, and anxiety [5,6]. The risks linked to corporal punishment include heightened risk of both perpetrating and being a victim of violence throughout one’s life [7]. A 2017 study found that experiencing corporal punishment in childhood was associated with mental and behavioural health impairments during adulthood, including increased odds of suicide attempts, increased drinking, and the use of street drugs [8]. Qualitative research by Saunders (2020) [9] found that children tend to experience corporal punishment as humiliating, intimidating, and frightening, all of which impair relationships and relate to poor mental health throughout the lifecourse. The Council of Europe describes corporal punishment as “the most widespread form of violence against children” [10].

Globally, the list of countries with legal prohibitions against all forms of corporal punishment continues to grow. At the time of submission of this article, 65 countries had prohibited corporal punishment in all settings and another 27 had declared an intention to do so [11]. Yet in Canada, section 43 of the Criminal Code still gives parents the right to use reasonable physical force, including corporal punishment, for disciplinary purposes. All forms of violence, including corporal punishment, violate children’s fundamental right to security and physical integrity as protected in the Convention on the Rights of the Child, which Canada ratified in 1989 [12,13]. The United Nations Committee on the Rights of the Child has repeatedly called for the repeal of section 43 and has expressed “grave concern” about Canada’s continued lack of action [14]. To date, 669 respected organizations have called for its repeal, including the Children’s Hospital of Eastern Ontario; many child welfare authorities across the country; Hospital for Sick Children (SickKids), Toronto; Canadian Medical Association; Canadian Mental Health Association; and Canadian Psychological Association [1]. In 2019, the Canadian Paediatric Society made a strong statement against any use of physical punishment in parenting [15]. Yet Canada’s law remains unchanged, with no commitment to future action. While public education efforts seem to have decreased the prevalence of corporal punishment in Canada, far too many children are still at risk. About 25% of Canadian parents (and 35% in a Quebec sample) use corporal punishment as a parenting strategy [16].

Corporal punishment remains the most common form of violence against children in Canada. Along with being legally permitted, it is made socially acceptable through cultural and social norms often disguised as discipline [17]. Paraphrases of Judeo-Christian sacred texts such as “spare the rod; spoil the child” have been widely used to justify corporal punishment, and to create norms around the idea that it is a god-given responsibility of parents to inflict corporal punishment on their children “for their own good.” Physical punishment is then not only an acceptable part of raising children but can be a godly duty. Because of Canada’s history of colonization, Christianity has played a dominant role in shaping norms and beliefs (see [18]). Though Canada is a secular country, colonial Christian social norms around violence provide a moral hegemony [19] under which violence becomes the status quo [20], and that has proven very difficult to change. As Beaman suggests, “despite a pervasive rhetorical commitment to religious diversity” ([21], p. 145), a form of “Christian privilege” [22] is protected in Canadian law. Furthermore, Euro-Christian beliefs are so subtly woven into the fabric of secular Canadian society that they often go unnoticed [23].

All the scientific evidence needed to demonstrate unequivocally that the use of corporal punishment poses serious risks to children has been known for some time. That its use is still so prevalent in Canada indicates a clear need for an approach to protecting children that extends beyond scientific research evidence about its harms. Historical Christian interpretations of sacred text have played a strong normalizing role in perpetuating the hitting of children for disciplinary purposes. Christian faith leaders have a strategic opportunity to change the harmful punitive narrative by challenging these deeply ingrained attitudes.

Christianity is certainly not the sole reason corporal punishment is prevalent. Yet the role of Christianity is important to consider because from antiquity, corporal punishment has been justified by religious texts and cultural beliefs, which often shape each other [24]. In Canada, Christianity is the dominant religion (in 2021, 53.3% of those surveyed reported a Christian religion [25]). It is a substantial contributing factor that, to date, has not been adequately addressed and countered. The purpose of this commentary is to focus on this specifically, in the context of a much broader movement to eliminate corporal punishment from the lives of children.

In this commentary, we outline the ways that Christian religion has contributed to social norms around corporal punishment. We then argue that religious leaders, and indeed parents, grandparents, caregivers, and anyone who identifies with the Christian faith, have an opportunity—and moral responsibility—to contribute to change. We conclude with insights from social norms theory and offer evidence-based recommendations for ways forward that could help shift attitudes around corporal punishment in order to decrease its prevalence and harm. While each of these issues has been written about extensively in isolation, because of the continued relationship between identification with the Christian religion and prevalence of corporal punishment, this commentary offers an analysis of these matters under one umbrella. By making this relationship between Christianity and the social norms that propagate corporal punishment more visible, we draw attention to the role that leaders in Christian churches could play in disrupting the social acceptance of violence against our youngest Canadian citizens.

This commentary is based substantially on the involvement of both authors in addressing this challenge. We have done this through concerted efforts over decades to advocate for faith groups to take seriously the well-being of children, including through inviting faith-based groups to endorse the Joint Statement on Physical Punishment of Children and Youth [1]; currently, several major Canadian faiths have endorsed the Joint Statement). Through our work, we are also encouraging Church parties to the Truth and Reconciliation Settlement Agreement to be accountable to the role that Christian theological messaging has played in normalizing violence against Indigenous children, in particular [18].

## 2. Social Norms, Corporal Punishment, and Christian Religion

Social norms are the unspoken rules or expectations of what is appropriate behaviour within a cultural group or society, and they are strong influencers of human behaviours. While they can be used to create social cohesion and a sense of belonging in a community, they can also be used to justify the violation of human rights and to maintain harmful and discriminatory practices [26]. In the case of corporal punishment, when it is endorsed by a religious community and supported by interpretations of sacred texts, a social tolerance of violence as a legitimate way of solving problems is established and provides a moral pretext for its use.

Research shows that people who profess the Christian faith are one of the main groups who support the use of corporal punishment [27,28,29]. Periodically, egregious examples of corporal punishment that have been used in Canadian church contexts are profiled in mainstream news. Illustratively, a recent example was reported in relation to Legacy Christian Academy in Saskatoon, where corporal punishment was used extensively. News reports make reference to a school training manual that not only advocates the use of corporal punishment, but appears to suggest that child psychologists who discourage corporal punishment have been influenced by “the devil” [30]. While it is easy for many to agree that high profile examples such as the use of corporal punishment at the Academy in Saskatoon are shocking and wrong, a seemingly tacit acceptance of violence against children remains. Christian religion—implicitly and explicitly—continues to be used to justify acceptance of corporal punishment as appropriate discipline.

This silence is troubling. When social norms around corporal punishment as a method of parenting go unchallenged, interpretations of Christian texts continue to be used to support violent punishment of children. Even though many faith leaders in Canada would quietly disagree with the use of corporal punishment, this silence allows for a tacit acceptance of everyday violence and abuse in the home. In turn, family patterns of violent punishment, which are often transmitted intergenerationally, sustain the harmful beliefs that corporal punishment is a normal and effective part of parenting for another generation [31].

Christian leaders should be concerned about this for many reasons. First, the research is clear and unequivocal: all corporal punishment puts children at risk for adverse health and developmental outcomes. Corporal punishment is simply bad for the health and adjustment of children. An impressive spectrum of Canadian [18,32] and international [33,34,35,36,37] theologians have argued that corporal punishment, too, is bad religion, and is inconsistent with a Christian biblical understanding of children and childhood.

Several explanations for the tacit compliance with corporal punishment exist. It may be that many are not aware that it remains so prevalent in Canada. It may also be that corporal punishment is so deeply normalized in Canadian society that it is not considered violent and harmful. It is also likely that many Christian community members who do not support corporal punishment do not understand the critical role they could play in changing this harmful social norm in Canada. They could support renorming Canadian society regarding violence against children, and in so doing they could promote providing children with the same protection from violence that is taken for granted by adults.

The international and multifaith initiatives to eliminate corporal punishment are impressive. In 2006, Religions for Peace and UNICEF convened a global, multifaith consultation to provide a religious perspective to the UN Secretary General’s Study on Violence against Children. One outcome was the declaration: “A Multi-Religious Commitment to Confront Violence against Children”. The declaration reads, “We find strong consensus across our religious traditions about the inherent dignity of every person, including children. This requires that we reject all forms of violence against children and protect and promote the sanctity of life in every stage of a child’s development” [38].

The declaration continues:

*We must acknowledge that our religious communities have not fully upheld their obligations to protect our children from violence. Through omission, denial and silence, we have at times tolerated, perpetuated and ignored the reality of violence against children in homes, families, institutions and communities, and not actively confronted the suffering that this violence causes. Even as we have not fully lived up to our responsibilities in this regard, we believe that religious communities must be part of the solution to eradicating violence against children, and we commit ourselves to take leadership in our religious communities and the broader society.*

Signed by religious leaders around the world, the declaration specifically includes protecting all children from corporal punishment as an urgent task.

There are many similar initiatives specifically rooted in the Christian religion. For example, in 2013, the document “Putting Children at the Centre” was drafted in Busan, Korea, at the 10th Assembly of the World Council of Churches. Here again, global church bodies made a commitment to take seriously the “everyday violence of corporal punishment” and made a commitment to “work with others in the global movement to prohibit and eliminate corporal punishment of children” [39]. This momentum continued. In 2017, the World Council of Churches and UNICEF partnered to develop a document called “Churches’ Commitments to Children”. Drawing attention to the resonances between Christian responsibilities towards children and children’s rights as expressed in the “Convention on the Rights of the Child” [40], this document articulates the alignment of a rights-based understanding of childhood and a theology of childhood that is rooted in love, compassion, and respect. The core commitments made here include: promoting positive parenting as a way of preventing violence against children in the home; challenging the attitudes and behaviours that underlie violence towards children; and working to end harmful “traditional practices that may be related to sociocultural and religious beliefs, including … violent discipline” ([40], p. 6). In Canada in 2016, a diverse group met to develop A Christian Theological Statement in Support of the Truth and Reconciliation Commission’s Call to Action No. 6, which was endorsed by church leaders from across Canada [18]. Each of these initiatives acknowledges the ways that Christian theological messaging has been used to normalize and justify violence against children and seeks to disrupt this narrative and replace it with a healthier theological message—one that protects children. Christian interpretations of sacred texts are not solely responsible for the idea that some forms of violence are not only normal and justifiable, but they are recognized by faith groups and secular scholars alike as being a substantial contributing factor.

Modifying these kinds of deeply ingrained social and cultural norms and behaviours is emphasized as one of the seven INSPIRE strategies for Ending Violence Against Children [41,42]. Canada’s 2016 pledge to be a “Pathfinder country” with the INSPIRE strategy (Global partnerships) came with a commitment to innovative leadership and action in addressing “drivers of violence” through integrated national responses (End Violence Against Children). Yet changing “harmful societal norms” (Inspire Strategy 2) has proven to be challenging. Even amid the many global and national initiatives to change the widely accepted narrative that justifies violence against children, in Canada these same social norms persist. In part, this is because in Canada religion is diverse and varied. While most of the perspectives we have presented in this commentary are universal in their agreement that corporal punishment has no place in the lives of children, not all religious leaders and other stakeholders would agree (e.g., see [43,44]). Diversity is one of the great strengths of Canadian society and of world religions. Yet, when religious views cause harm to the well-being of children, they need to be challenged and disrupted. While respecting principles of freedom of religion, we argue that there is no room for diversity around the belief that children deserve full protection from violence. Because of the history of the way sacred texts have been used to create and sustain cultures that normalize violence, many scholars and advocates have called for religious groups to become involved in addressing norms around corporal punishment [27,29,45].

## 3. A Framework for Changing Social Norms around Corporal Punishment in Canada

Social norms can and do change. Examples of successful efforts to change social norms include issues such as infant car seats and child seat belts, no smoking zones, impaired driving, and helmet wearing. While societal changes usually happen slowly and in stages, attitudes towards these behaviours have changed relatively dramatically, due largely to intentional education initiatives, public awareness campaigns, and legislation (e.g., [17,46,47]). There is not one overarching formula changing social norms, but research suggests multifaceted strategies that utilize credible messengers from within the targeted group are crucial. The interdependence between increased awareness and legislation and/or policy change is also clear. Changes in attitudes can be a catalyst for changes in law, while changes in law can also be a catalyst in changing attitudes and behaviours [48,49].

Christian leaders in Canada could play a strategic role in addressing the normalization of violence in church contexts and providing a counter narrative that supports the flourishing of all children. This could hasten a “tipping point” [50]—a point at which a critical mass of relevant actors adopts the new norm. In turn, this can trigger a “norm cascade” [51,52,53] in which wide acceptance of a new norm begins to ripple through the wider population. 

There is considerable research that demonstrates the strength of this approach. Illustratively, the same process we propose to change norms around corporal punishment has also been used to address other harmful behavioural patterns using this “tipping point” theory. This includes intentional action to change norms around smoking [54,55], driving without seatbelts, failure to use child car seats [46], and norms around female genital mutilation [56]. What each of these examples illustrates is that an approach to change that addresses social norms can make a strategic and efficient contribution to creating large-scale changes in social expectations and behaviours that are harmful.

A coherent, faith-rooted strategy to change norms around corporal punishment in Canada could learn from other successful initiatives (such as no smoking zones and seatbelt campaigns) and include (1) creating education and training opportunities; (2) implementing public awareness campaigns; and (3) supporting legal reform. This strategy has the potential to hasten movement towards a public and institutional “tipping point” around corporal punishment by bringing religious leaders on board. All the evidence and theological arguments that should be needed to eliminate corporal punishment already exist. What is needed is more tangible action to change deeply embedded social norms. In the next three subsections we provide information and guidance about each of these three evidence-based strategies we have proposed in order to effect this change. 

While this approach is not dependent on support, or “buy-in” by clergy and other religious leaders, it is certainly strengthened by their support. Here again, the strategy of “credible messengers” from within a group may be useful. Religious leaders may be much more open to this message when it comes from leaders from within one’s own denomination. In Canada and internationally, a diverse range of theologians and denominational leaders have spoken in support of fully protecting children from violence and eliminating corporal punishment. Their messages provide targeted starting points for “in-house” (or “in-denomination”) discussions as further initiatives are planned. (See [57], for a global perspective on the ways that religious communities around the world are addressing corporal punishment and encouraging law reform. This chapter points to statements from the Southern African Catholic Bishops’ Conference; the Caribbean Coalition for the Abolition of Corporal Punishment of Children; the Church of Scotland General Assembly; and Anglican Bishops, New Zealand).

### 3.1. Education and Training

Research globally demonstrates that education is critically important to changing social norms and supporting positive parenting practices. Table 1 presents a summary of resources that are specifically geared to help faith communities with this task. This repository includes theological and biblical analyses of corporal punishment and child discipline; worship resources; and other resources that can be used to develop strong educational programs and to advocate for a positive approach to discipline. These are not universally transferable across faith groups, nor are they necessarily relevant beyond faith groups. Many resources are available that inform this social issue from the perspective of religions globally and also from nonreligious perspectives.

In Table 1, we have also included resources that address gender-based violence from a faith perspective because the same social norms that normalize violence against children are often rooted in patriarchal ideas of gender roles, power, and control: attitudes that are often amplified through Christian interpretations of sacred texts. Even though corporal punishment is the most prevalent form of violence against girls globally, it is often overlooked in discussions of gender-based violence [11]. Yet, the use of corporal punishment often follows gendered patterns, and is used to control girls’ “social and sexual behaviour, to encourage deference, submission, and timidity, or to reinforce traditional ideas of what it means to be a woman” ([11], p. 1). Experiencing corporal punishment as a child increases the risk that girls will experience intimate partner violence later in their lives. It is not surprising then, that the Global Initiative identifies the elimination of corporal punishment as “essential in preventing violence against women and girls—both directly and as part of a broader strategy for eliminating other forms of violence” ([11], p. 1). Just as religious leaders could help to shift social discourses around the normalization of violence against children, they could also use their influence to provide moral and theological leadership in changing social norms around harmful gendered attitudes and behaviours. 

A promising and cost-effective strategy that may be useful in church contexts where budgets cannot support large-scale interventions, is an approach called “organized diffusion” [58]. Phase 1 involves discussions before an educational program is launched. As rumours of the upcoming intervention program circulate, curiosity begins to be generated from within the community. In phase 2, participants in the actual program gain new knowledge and skills, which they intentionally share with one other community member in phase 3. In the remaining three phases, information spreads from the intervention community to new communities and networks, who are engaged in transformative conversations [58]. While this approach has not been widely evaluated, initial case study research demonstrates its potential to shift harmful social norms and be a catalyst for transformative conversations within communities.

Educative approaches are generally most effective when used in combination with training—facilitating the opportunity for people to practice alternative approaches and behaviours that reflect new, more positive norms. A promising group-based parenting program, Positive Discipline in Everyday Parenting [59], developed through a collaboration between a Canadian academic researcher and the global nonprofit child rights organization Save the Children, has been demonstrated to be highly effective, and has clear potential for use in faith settings. The eight-week curriculum guides parents in understanding the reasons that tend to underlie parent–child conflicts and helps parents to implement nonpunitive problem solving in keeping with children’s social, emotional, and brain development [59].This program could be run in church contexts, and could target parents, grandparents, and all adults who care for children as initial priority populations. Further, it could be used in conjunction with some of the strong theological literature that separates discipline from punishment and provides a rich theological framework for positive child discipline [34].

Church leaders are another important target subgroup for education initiatives. Many parents look to clergy and faith leaders as having a kind of moral authority around child rearing [28,29,45]. When a religious leader is not conversant with child development and relies instead on deeply embedded social norms around scriptural passages that appear to support corporal punishment, it is much more likely they will recommend corporal punishment as a parenting tool [28]. The risk that these parents will use corporal punishment in their own families thus increases. This may partially explain what Taylor et al. (2013) [60] found—that parents who seek advice from religious leaders about child discipline are four times more likely to employ corporal punishment than those who seek parenting advice from a paediatrician. Seminary education could include renorming around the role of discipline and training in positive approaches to discipline. Graduated seminary students would then be equipped to support parents in ways that are theologically and evidence-informed. In some denominations, parents look to religious leaders for guidance on raising their children in accordance with their faith tradition as the rite of baptism approaches. Baptismal education programs may also offer a strategic opportunity for integrating a rich theological understanding of childhood that also includes training in positive discipline.

### 3.2. Public Awareness Campaigns

Public awareness campaigns can be used to draw attention to the negative consequences of harmful behaviours, and also to reframe harmful behaviours that have previously been accepted as normal. This process can be thought of as “renorming” [51]. The theory behind renorming suggests that when individuals mistakenly perceive others are behaving in a risky or negative way, that behaviour is nonetheless normalized, and they are much more likely to engage in it as well [51]. When the message of “everyone is doing it, so it’s ok” is no longer normalized, the social tolerance or acceptance of the behaviour decreases. A “renorming campaign” that draws attention to the number of theologians, advocates, and church leaders who have spoken against corporal punishment could be highly effective as one piece of the strategy to change attitudes towards corporal punishment. These campaigns could be launched at both individual parish and denominational levels. This change process has already been demonstrated by the growing number of Canadian faith groups that have endorsed the Joint Statement on Physical Punishment of Children and Youth [21].

Churches have unique opportunities to launch renorming campaigns through regular notes in church bulletins (see Table 1), and advertisements, interviews, guest presentations and sermons, book reviews, and editorials in faith-based newspapers and magazines. Worship services can be a highly effective way of replacing the social normalization of violence with a new social norm: that violence against children in the form of corporal punishment has no place in parenting or in Christian life.

### 3.3. Active and Vocal Support of Law Reform

As critical as education and public education campaigns are, in isolation they are less effective because section 43 of the Criminal Code undermines educational messaging related to the enduring personal and societal harms linked to physical punishment of children. Repeal of section 43 is essential to providing children with the same protection from assault that is taken for granted by adults [48]. Only parliamentary repeal can strike this ancient statute from Canadian law. Repeal of section 43 would be a tangible way for society to express its pledge to the protection of children.

The purpose of repeal is first and foremost to protect children and uphold their human rights. Secondly, bans can be used to change attitudes and behaviours and send a strong message across society that it is never okay to hit a child ([26], p. 1). While bans do not result in instant universal behavioural change, they do drive changes in attitudes and behaviours [26,61]). The intention of such a ban is educative—not punitive. International comparisons show that this approach is highly effective in decreasing the use of corporal punishment without criminalizing parents [24].

A strength of the multidimensional approach we have proposed is that it advocates for using three distinct approaches to the same message. It is rooted in research demonstrating that the strongest impact in protecting children from violence is achieved when legal reform is used in combination with education and public awareness campaigns [62].

To date, Christian groups have provided some of the strongest endorsement of corporal punishment in Canada (e.g., [63,64,65]). Yet, by concretely addressing the harmful social norms around violence within their own faith contexts, Christian leaders also have the potential to add momentum to the movement to eliminate corporal punishment in Canada. This can be done through education and awareness and becoming active and vocal supporters of repeal.

## 4. Strengths and Limitations

While each of the issues we have discussed in this commentary have been written about extensively, they have rarely been considered together under one umbrella. A strength of this commentary is that by illuminating the relationship between Christianity and the social norms that propagate corporal punishment, we draw attention to the role that leaders in Christian churches could play in disrupting the social acceptance of violence against children. We provide a practical and evidence-based framework, rationale, and appeal to Canadian faith leaders to use their voices to contribute momentum to a critical health, rights—and religion—issue in Canada.

Our commentary also has limitations. While the focus in this commentary is on the Christian religion, Christianity does not have a monopoly on the normalization of violence. Religious leaders of all faiths have a responsibility to address the ways that violence disguised as discipline has been supported by interpretations of various sacred texts from a wide range of traditions [66,67]. Our focus is on leaders and other stakeholders in Christian churches because of the influence that colonial Christianity has had on shaping British Common Law and on resultant societal social norms. Further, because of Canada’s relatively young history of colonization, Christianity has had longer to shape the normalization of violence against children in Canadian society than many other religions. A more complete analysis of the role that religion has played in shaping cultural norms around violence—including an analysis of the positive initiatives happening globally to disrupt harmful norms—would include a broader range of religions.

The educational approaches and interventions that would integrate theological messaging with evidence-based positive parenting programs we have proposed have not, to our knowledge, been rigorously evaluated in church contexts. Yet, related research gives us cause for optimism. Illustratively, recent research regarding changing social norms around female genital cutting in West Africa engaged religious and community leaders as a key part of a strategy for change that is similar to what we have proposed in this commentary. Here, researchers demonstrated the “transformational power” of such community-led processes to support people in changing norms and associated behaviour and in broader transformation [68].

## 5. Conclusions

The goal of this commentary is to draw attention to the strategic opportunity—and responsibility—stakeholders within the Christian faith have to challenge and change harmful social norms around child violence in Canada. Corporal punishment puts all children at risk for adverse health and developmental outcomes through the lifecourse. While there has been progress, religious communities continue “to be associated with opposition to prohibition and some pose a serious obstacle to legal reform” [35]. Social norms theory is helpful in that it expands existing understandings of why parents choose to use corporal punishment in their parenting. It further provides insights into ways to disrupt harmful social norms. Understanding and then reforming norms that sustain the practice of corporal punishment have the potential to increase the effectiveness of interventions.

A multidimensional evidence-based strategy that focuses on the disruption of social norms which rationalize corporal punishment to shift what is seen as “normal” and “acceptable” is badly needed in Canada. Globally, many religious leaders from across faith traditions have taken up the cause of protecting children from violence. In Canada to date, action in this area has been slow. Church leaders have an opportunity and responsibility to immediately stop normalizing, justifying, or turning a blind eye to violence against children as having any kind of legitimate place in Christian faith—or in any other arena. This will include publicly and vocally challenging communal social norms that condone violence as an acceptable way to resolve conflicts, regulate behaviours, and determine how individuals and groups should treat one another.

Canadian children remain vulnerable to violence in their own homes that is both legally protected and socially normalized. Addressing the social norms that sustain the practice of corporal punishment must be a coherent and sustained strategy that concretely addresses violence directed at children and reconstructs society in the best interests of all children.

## Figures and Tables

**Table 1:** Faith-based resources for addressing corporal punishment and gender-based violence.

Resource Name	Authors	Description	URL/Reference
Focus on corporal punishment			
A biblical and theological basis for ending violence against children	Author: John Baxter-Brown in partnership with World Vision and It takes a world to end violence against children	This resource provides a Christian biblical rationale for ending violence against children.	https://www.oikoumene.org/sites/default/files/File/Theological%20Narrative.pdf (accessed on 13 December 2022)
Churches’ commitments to children: Churches uniting for children in the pilgrimage of justice and peace	World Council of Churches and UNICEF	This resource was developed to strengthen action with and for children by church partners. It provides a biblically grounded commitment that is also rooted in the Convention on the Rights of the Child and the Sustainable Development Goals. It provides concrete ideas of things that faith leaders can implement to support, protect, and honour the contributions of children, including addressing the “everyday violence of corporal punishment” and gender-based violence.	https://www.oikoumene.org/resources/documents/churches-commitments-to-children (accessed on 13 December 2022)
Churches’ Network for Nonviolence	A Christian statement supporting legislation to end corporal punishment of children	This theological statement supports legislation to end corporal punishment of children from a faith perspective.	http://churchesfornon-violence.org/wp/wp-content/uploads/2012/04/A-statement-supporting-legislation-to-end-corporal-punishment-of-children-2012-J.pdf (accessed on 13 December 2022)
Ending corporal punishment of children—a handbook for worship and gatherings	Churches’ Network for Non-violence; Global Initiative to End All Corporal Punishment of Children	This resource includes multiple Bible study guides and reflections; vigil, prayer, and other liturgical resources; opportunities for eliminating corporal punishment in church settings; and opportunities for action. It also includes a section on ending domestic violence and a glossary of other relevant resources.	https://jliflc.com/wp-content/uploads/2020/02/Ending-corporal-punishment-of-children-A-handbook-for-worship-and-gatherings.pdf (accessed on 13 December 2022)
Faith-based support for prohibition and elimination of corporal punishment of children—a global overview.	Churches’ Network for Non-violence	This resource provides a summary of the growing number of faith-based initiatives to eliminate corporal punishment globally. It includes examples of religious communities working in collaboration with secular organizations, bound by their mutual commitment to children’s rights.	https://jliflc.com/resources/faith-based-support-for-prohibition-and-elimination-of-corporal-punishment-of-children-a-global-overview/ (accessed on 13 December 2022)
Ending Corporal Punishment of Children: a handbook for working with and within religious communities	Global Initiative to end All Corporal Punishment of Children; Save the Children Sweden; Churches’ Network for Non-violence	This multifaith resource provides an analysis of corporal punishment using religious texts and scholarly work, and also draws on the United Nations Declaration of Human Rights. It provides insights for addressing faith-based opposition to the elimination of corporal punishment and a call for religious leaders to take action.	https://resourcecentre.savethechildren.net/pdf/4420.pdf/ (accessed on 13 December 2022)
Joint Learning Initiative on Faith and Local Communities: Strengthening Evidence-Based Faith Engagement	Ending Violence Against Children Hub	This web-based hub houses multiple resources that address violence against children, women, and girls from a faith lens. Many of the resources seek to engage faith-based actors in preventing violence. It also includes resources related to masculinities, faith, and peace.	https://jliflc.com/resources/?_hub=evac (accessed on 13 December 2022)
Joint Learning Initiative on Faith and Local Communities: Strengthening Evidence-Based Faith Engagement	Gender-based violence learning hub	This web-based hub houses many resources that address violence against children, girls, and women, including intimate partner violence. Many of these resources advocate for faith leaders to engage in “norms-shifting” interventions.	https://jliflc.com/resources/?_hub=gender-based-violence (accessed on 13 December 2022)
Multiple authors, in: Michaelson and Durrant, 2020	A Christian Theological Statement in Support of the Truth and Reconciliation Commission’s Call to Action #6	Theological statement that specifically addresses the repeal of section 43 in the context of Call to Action 6 of the Truth and Reconciliation Commission of Canada.	https://dq5pwpg1q8ru0.cloudfront.net/2022/03/08/05/12/01/5e0b52cf-4703-418b-9f27-65f8ba2d9d79/Theological-Reponse-to-Call-to-Action-6-20171024-FINAL.pdf (accessed on 13 December 2022)
Michaelson and Durrant, editors, 2020	Decolonizing discipline: Children, corporal punishment, Christian theologies, and reconciliation. Univ. of Manitoba Press, 2020	The chapters in this edited volume address the repeal of section 43 in the context of Call to Action 6 of the Truth and Reconciliation Commission of Canada. It includes reflections by many First Nations, Métis, and Inuit rights-holders, along with Canadian church leaders and theologians.	Not available online [18]
Multireligious commitment to end violence against children: Kyoto Declaration, a 10th anniversary guide for reflection and discussion	Save the children; Global Initiative to End All Corporal Punishment of Children; Churches’ Network for Non Violence	This resource provides a guide for reflection and discussion on the Kyoto Declaration. For use in church and/or multifaith contexts. The Kyoto Declaration is included.	http://churchesfornon-violence.org/wp/wp-content/uploads/2016/11/Kyoto-Declaration-Guide.pdf (accessed on 13 December 2022)
Webb, W., 2011	Corporal punishment in the Bible: A redemptive-movement hermeneutic for troubling texts. InterVarsity Press, 11 July 2011.	Webb is a Canadian theologian who addresses corporal punishment by confronting what he calls the “troubling texts” that have been used to endorse corporal punishment in many church contexts.	Not available online [32]
Focus on gender-based violence			
Because God Loves Me—Affirming My Value in Christ	Yvette A. Kelem and Blandine E. Ackla, Editors	This Christian education program (written in French) is a resource that is designed to help children address gender-based violence (ages 1–12 years)	https://www.oikoumene.org/resources/publications/parce-que-dieu-maime-affirmer-ma-valeur-en-christ (accessed on 13 December 2022)
Gender Justice Principles with Code of Conduct	World Council of Churches	This resource provides a set of Gender Justice Principles that are designed to promote “mutual and accountable gender just relationships” in the life of all World Council of Churches work.	https://www.oikoumene.org/sites/default/files/2022-03/Gender%20Justice%20Principles%20Web.pdf (accessed on 13 December 2022)
Ending Violence	Women’s Inter-Church Council of Canada	This website provides resources that are rooted in faith and address gender-based violence.	https://wicc.org/restore/resources/book-recommendations/ (accessed on 13 December 2022)
Gender-Based Violence	Evangelical Lutheran Church in America	This website provides links to many initiatives and resources that are being used to address gender-based violence, including human trafficking.	https://www.elca.org/Our-Work/Publicly-Engaged-Church/Justice-for-Women/Social-Issues/Gender-Based-Violence (accessed on 13 December 2022)

## Data Availability

Not applicable.
